# Isolated Cone Dystrophy With Secondary Macular Involvement Mimicking Vascular Insult and Optic Atrophy: A Case Report

**DOI:** 10.7759/cureus.103601

**Published:** 2026-02-14

**Authors:** Divya E Varghese, Stephen Sudhakar

**Affiliations:** 1 Ophthalmology, Sri Ramachandra Institute of Higher Education and Research, Chennai, IND

**Keywords:** cone dystrophy, electrophysiology, erg, full-field electroretinography, macular involvement, multimodal imaging, optic atrophy, retinal dystrophy

## Abstract

Isolated cone dystrophy is a rare retinal degenerative disorder characterized by selective dysfunction of cone photoreceptors, with relatively preserved rod function. Clinical presentation can be variable and, in rare cases, may mimic retinal vascular injuries or optic neuropathies, leading to diagnostic uncertainty. We report the case of a 32-year-old male patient with gradual, painless, asymmetric vision loss predominantly affecting the left eye. Fundus examination revealed optic disc pallor, arteriolar narrowing, macular pigmentary changes, and absence of the foveal reflex, raising initial concerns for retinal vascular insufficiency or optic atrophy. Multimodal imaging showed widespread macular thinning, photoreceptor layer disruption, and an enlarged foveal avascular zone, complicating the diagnostic evaluation. Full-field electroretinography demonstrated preserved scotopic responses but markedly reduced photopic responses, confirming selective cone dysfunction and establishing a diagnosis of isolated cone dystrophy with secondary macular involvement. The absence of systemic vascular risk factors, a routine neurological examination, and characteristic electrophysiological findings were pivotal in differentiating this condition from vascular or optic nerve disorders. This case highlights the importance of considering cone dystrophy in the differential diagnosis of painless, progressive vision loss with optic disc pallor and macular changes. It underscores the critical role of electrophysiology and multimodal imaging in accurate diagnosis.

## Introduction

Isolated cone dystrophy is an uncommon, heterogeneous group of inherited or sporadic retinal degenerative disorders marked by selective impairment of cone photoreceptors, while rod function remains relatively intact [1]. The retina is a specialized neurosensory tissue lining the posterior segment of the eye and is responsible for converting light stimuli into neural signals that are transmitted to the visual cortex for visual perception. Cones are essential for central vision, color discrimination, and photopic (daylight) vision; they are predominantly concentrated in the macula and fovea, where high-resolution visual processing occurs [1-3]. Their degeneration primarily causes reduced visual acuity, color vision deficits, and photophobia, whereas scotopic vision usually remains unaffected in early and middle stages. Cone dystrophy can present at any age, from childhood to adulthood, and typically progresses over time, although the rate varies among individuals [2].

Clinically, cone dystrophy manifests as diminished central vision, color vision impairments, and increased susceptibility to glare. These deficits often interfere with visually guided tasks such as reading, facial recognition, and fine-detail discrimination, and may lead to subjective visual discomfort or visual fatigue [2]. Fundus findings may be subtle or normal in early stages, whereas advanced stages may show macular atrophy, retinal pigment epithelium (RPE) mottling, optic disc pallor, and thinning of retinal capillaries [3]. As the disease progresses, secondary macular involvement becomes more apparent, leading to permanent central vision loss [4,5]. These structural changes often resemble features of retinal vascular disorders or optic neuropathies, complicating diagnosis.

Electrophysiological testing, particularly full-field electroretinography (ERG), is essential for diagnosis. ERG provides an objective assessment of retinal function that is independent of patient perception or cooperation [5-7]. Typical findings include markedly reduced or absent photopic responses, with preserved scotopic responses, confirming selective cone dysfunction [6]. Pattern ERG and multifocal ERG can further delineate the involvement of macular and central cone pathways. Advances in multimodal retinal imaging, including optical coherence tomography (OCT) and fundus fluorescein angiography (FFA), have enhanced understanding of structural changes, such as outer retinal layer thinning, disruption of the ellipsoid zone, enlargement of the foveal avascular zone, and macular atrophy [7,8].

Cone dystrophy may follow autosomal dominant, autosomal recessive, or X-linked inheritance, although sporadic cases also occur. Mutations in genes regulating phototransduction, cone outer segment formation, and retinal metabolism have been implicated [9]. Despite advances in genetics, significant phenotypic variability exists, even among patients with identical genotypes, which may influence both objective visual function and subjective visual symptoms. This variability can lead to delayed diagnosis or misclassification, especially in patients with atypical symptoms such as photophobia or isolated color vision impairment [10].

A major diagnostic challenge is that isolated cone dystrophy can mimic other ocular disorders, particularly retinal vascular insults and optic neuropathies. Clinicians may consider central retinal artery occlusion, anterior ischemic optic neuropathy, or compressive optic neuropathy when optic disc pallor, arteriolar narrowing, macular alterations, and visual field defects are present. In the absence of systemic risk factors or confirmatory testing, such misdiagnoses may result in inappropriate management and delayed detection of the underlying retinal disease [11,12].

Reporting cases of isolated cone dystrophy with atypical presentations is crucial for increasing clinician awareness of this diagnostic masquerade. Emphasizing the role of multimodal imaging and electrophysiological testing highlights the need for comprehensive retinal assessment in patients with unexplained vision loss and optic disc pallor. Case-based evidence improves diagnostic accuracy, enables timely prognostic counseling, prevents unnecessary procedures, and supports appropriate long-term follow-up. This work underscores the importance of considering cone dystrophy in the differential diagnosis of progressive, painless visual impairment and clarifies overlapping features with vascular and optic nerve pathologies.

## Case presentation

A 32-year-old male patient presented with a six-month history of progressive, painless visual deterioration in the left eye. The vision loss was gradual and initially unnoticed until it became more pronounced. The patient reported no photophobia, nyctalopia, metamorphopsia, ocular pain, redness, trauma, or prior ocular surgery. There was no history of systemic illnesses, including diabetes mellitus, hypertension, cardiovascular disease, or autoimmune disorders. The patient denied the use of medications known to affect retinal health and reported no family history of hereditary retinal disorders or optic neuropathies.

Systemic and general physical examinations were unremarkable, with stable vital signs and no neurological or systemic vascular abnormalities. Ophthalmic examination revealed best-corrected visual acuity of 6/6 in the right eye and hand movements near the face in the left eye. Intraocular pressure, measured by applanation tonometry, was 13 mmHg in the right eye and 12 mmHg in the left eye. A relative afferent pupillary defect was present in the left eye. Anterior segment examination was regular in both eyes: the cornea was clear, the anterior chamber was deep and quiet, the iris appeared normal, and the crystalline lens was clear.

Fundus evaluation of the right eye demonstrated temporal optic disc pallor, mild arteriolar narrowing, and fine granular pigmentation at the posterior pole. The left eye exhibited pronounced waxy pallor of the optic disc, extensive retinal arteriole constriction, a bronze-beaten appearance of the macula, absence of foveal reflex, mottling of the retinal pigment epithelium, and granular pigmentary changes.

**Figure 1:**
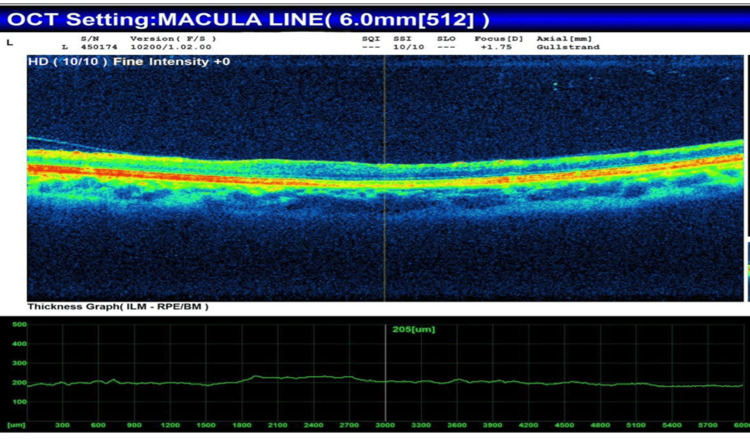
OCT of left eye showing diffuse macular thinning and partial PVD with loss of foveal contour OCT: optical coherence tomography; PVD: posterior vitreous detachment

OCT of the left eye revealed extensive macular thinning, disruption of the photoreceptor layer, partial posterior vitreous detachment, and altered foveal contour (Figure 1).

**Figure 2:**
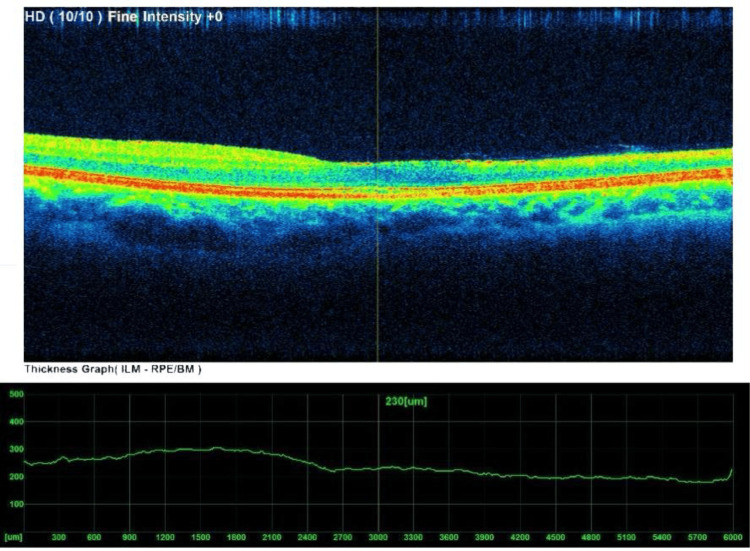
OCT of right eye showing papillomacular bundle narrowing nasal to fovea OCT: optical coherence tomography

OCT of the right eye showed thinning of the papillomacular bundle on the nasal side of the fovea, macular thinning, and early disruption of foveal architecture (Figure 2).

**Figure 3:**
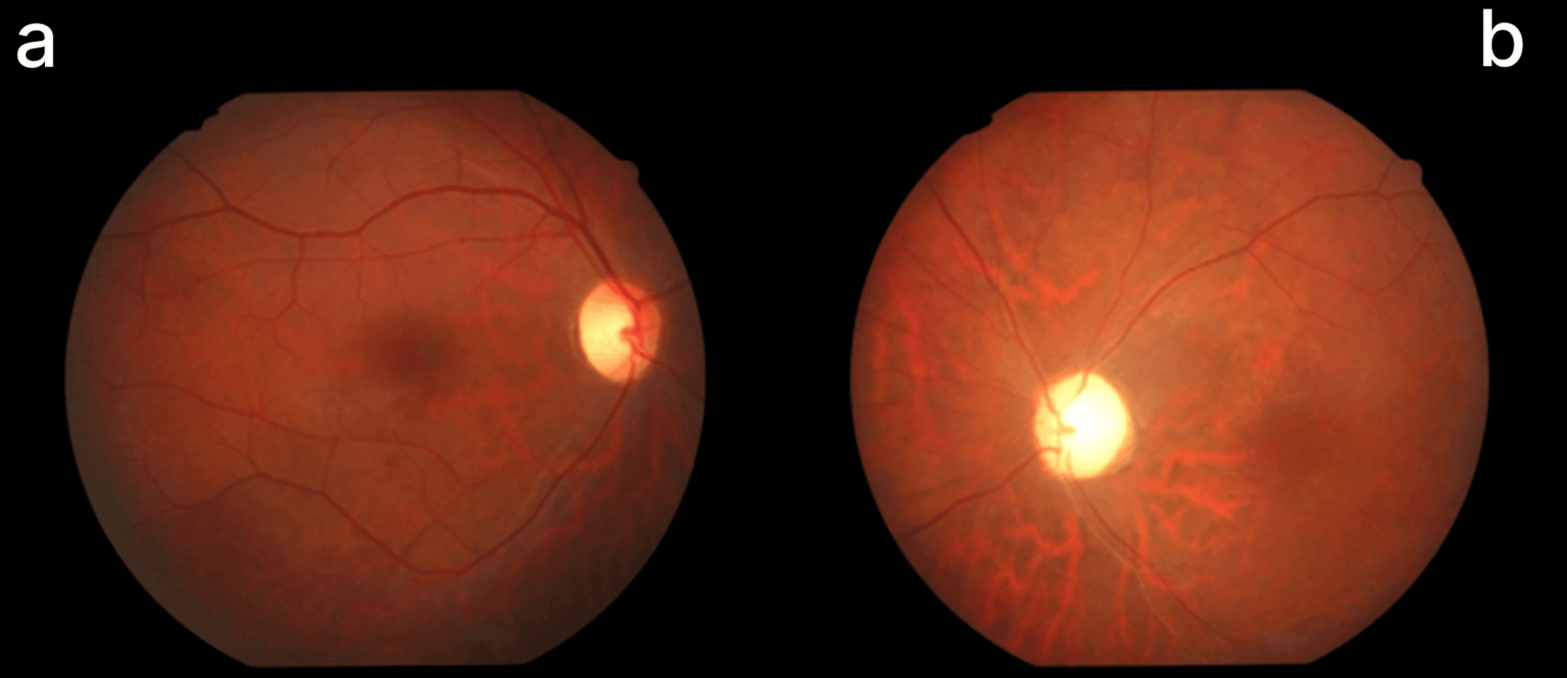
Fundus photos showing bilateral disc pallor in right eye (a) less than left eye (b), with narrowing of arterioles

**Figure 4:**
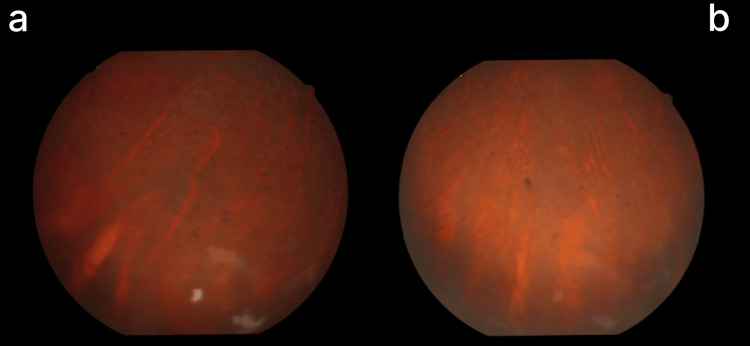
Fundus photo of right (a) and left (b) eye showing specs of pigments at peripheral retina

**Figure 5:**
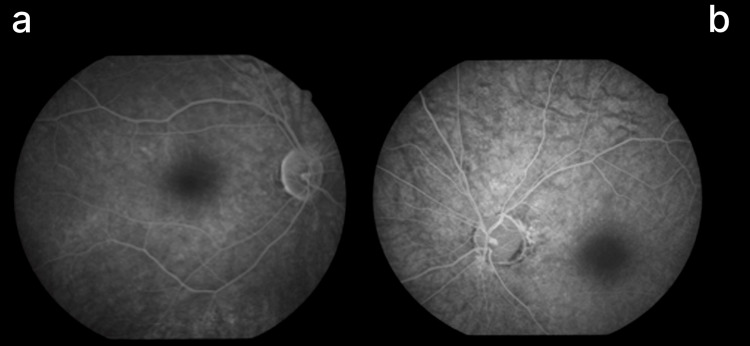
Fundus fluorescein angiography (a) right eye showing reduced caliber of artery and veins inferior > superior; (b) left eye showing generalized narrowing of blood vessels

FFA demonstrated granular hypofluorescence, widespread retinal arterial constriction, and an enlarged foveal avascular zone exceeding 1 mm in the left eye. In contrast, the right eye showed reduced vessel caliber, predominantly in the inferior region (Figures 3-5).

**Figure 6:**
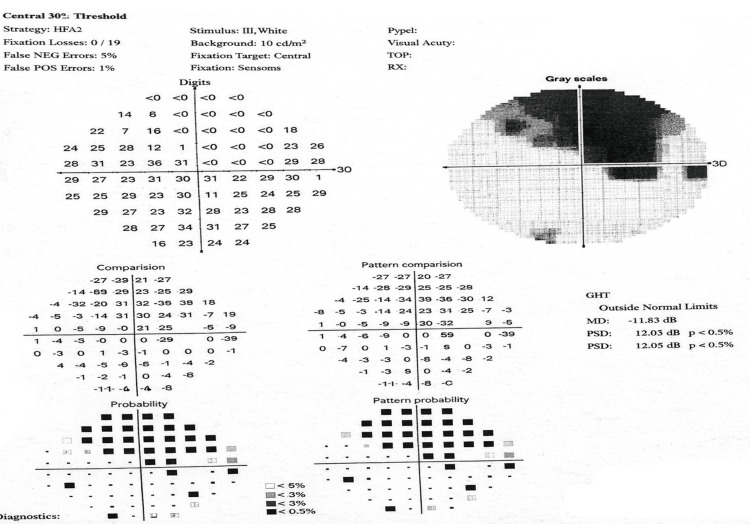
Automated visual field analysis revealing areas of visual field loss

Automated perimetry revealed an altitudinal visual field defect in the right eye; reliable visual field testing in the left eye was not possible due to poor fixation. Comprehensive vascular and neurological assessments, including systemic evaluation, were within normal limits (Figure 6).

**Figure 7:**
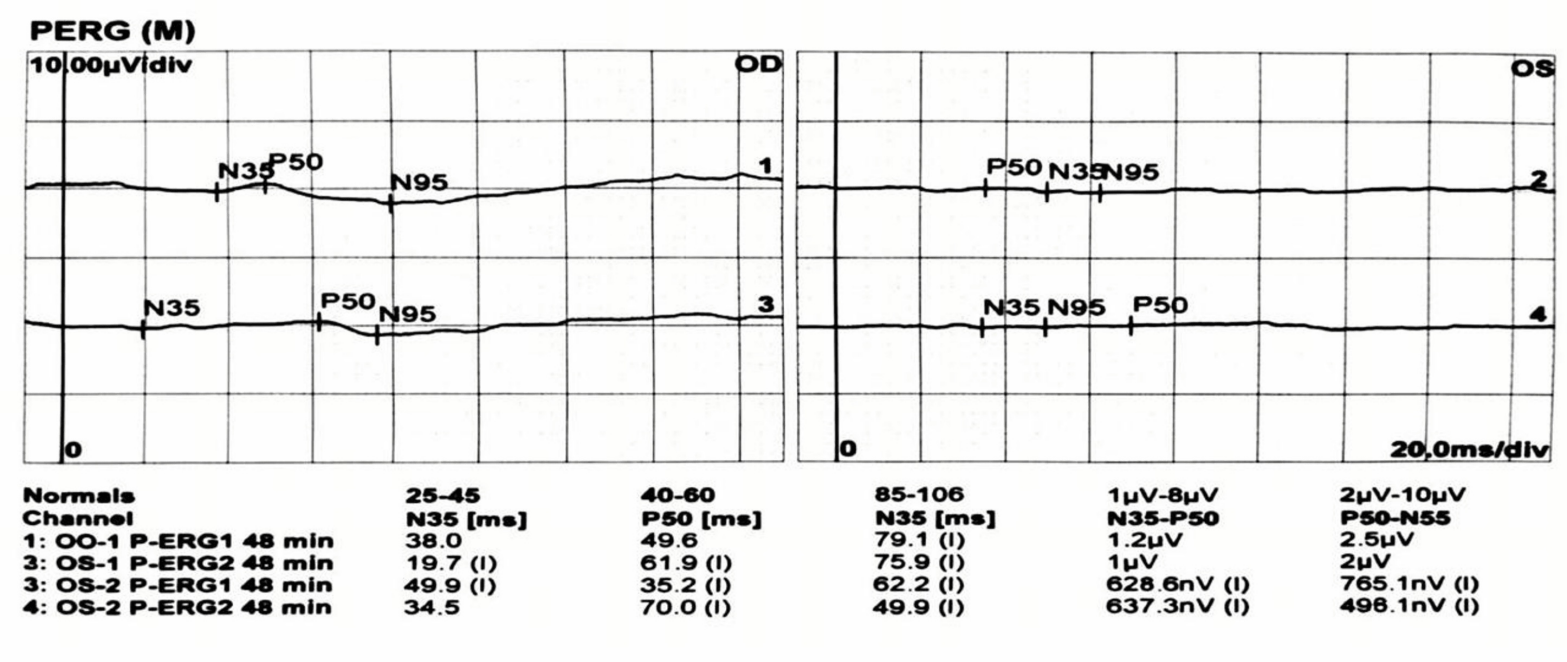
Pattern electroretinography (PERG) recordings showing N35, P50, and N95 waveforms in both eyes, reflecting retinal ganglion cell and inner retinal function

**Figure 8:**
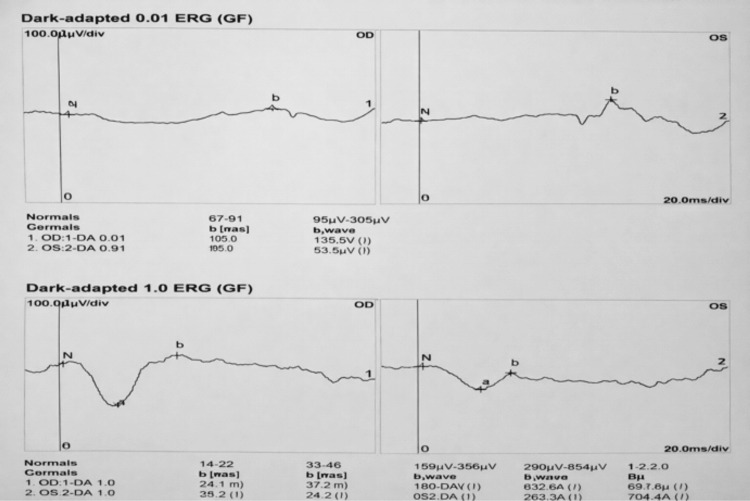
Dark-adapted electroretinography (ERG) responses at 0.01 and 1.0 cd·s/m² demonstrating rod-mediated retinal function in both eyes

Full-field ERG demonstrated normal scotopic responses bilaterally, indicating intact rod function. Photopic responses, however, showed attenuated a- and b-wave amplitudes, reduced 30-Hz flicker responses, and decreased P50 amplitude on pattern ERG, consistent with selective cone dysfunction (Figures 7, 8).

Based on the clinical presentation, multimodal imaging, and electrophysiological findings, a diagnosis of isolated cone dystrophy with secondary macular involvement was established.

## Discussion

This case demonstrates an unusual and diagnostically challenging presentation of isolated cone dystrophy with secondary macular involvement, closely mimicking retinal vascular insufficiency and optic atrophy. The early onset, asymmetric involvement, absence of classic symptoms such as photophobia or color vision deficits, and the presence of optic disc pallor with vascular attenuation initially suggested vascular or optic neuropathic etiologies. However, careful integration of multimodal imaging and electrophysiological assessment ultimately identified selective cone dysfunction as the primary pathology.

Cone dystrophy is typically characterized by progressive central vision loss with preserved rod-mediated scotopic function. In our patient, preserved scotopic ERG responses alongside selective attenuation of photopic a- and b-waves, reduced 30-Hz flicker, and decreased pattern ERG P50 amplitude provided conclusive evidence of cone-specific dysfunction. Georgiou et al. (2024) highlight this electrophysiological signature, emphasizing ERG as the cornerstone for differentiating cone dystrophy from optic neuropathies and retinal vascular disorders, particularly when fundoscopic features overlap [13]. The superior altitudinal visual field deficit in the right eye further complicated the clinical picture, as such defects are typically associated with ischemic optic neuropathy. Georgiou et al. note that secondary macular and papillomacular bundle involvement in cone dystrophy may produce deceptive field abnormalities, underscoring the importance of correlating structural and functional findings [13].

Retinal changes in our patient, including a bronze-beaten macula, an absent foveal reflex, macular thinning, and an enlarged foveal avascular zone, closely resembled those of chronic macular ischemia. Bauwens et al. (2024) provide a genetic and phenotypic framework explaining this vascular mimicry, showing that bi-allelic SAMD7 mutations can lead to selective foveal cone loss, perifoveal atrophy rings, mottled pigmentation, and bull’s-eye maculopathy on OCT, despite preserved rod function and absence of proper vascular occlusion [14]. Although genetic testing was not performed in our patient, the clinical similarities were striking: selective cone loss, preserved scotopic ERG, retinal thinning, and angiographic hypofluorescence without leakage. Bauwens et al. further emphasize that human foveal specialization renders cones especially susceptible to genetic alterations, explaining why these patients may be misdiagnosed with vascular maculopathies [14].

FFA revealed diffuse arterial constriction and an expanded foveal avascular zone, particularly in the left eye, initially suggesting retinal ischemia. However, Georgiou et al. note that secondary perfusion changes on OCT angiography and FFA can result from photoreceptor degeneration rather than primary vascular pathology [13]. Similarly, Shen et al. (2024) demonstrated that reduced cone density correlates with macular structural and blood flow changes, supporting the concept that cone loss can induce perfusion alterations independent of primary vascular disease, even though their study focused on myopia [15].

Optic disc pallor further raised concern for optic atrophy. Nevertheless, the absence of neurological signs, regular systemic evaluation, and preservation of inner retinal layers, in contrast to outer retinal damage, argues against primary optic neuropathy. This differentiation is critical compared with disorders such as RTN4IP1-associated recessive optic atrophy, in which optic atrophy precedes rod-cone dystrophy, ERG shows rod involvement, and widefield autofluorescence reveals peripheral retinal changes, features absent in our patient [11]. Likewise, Jouda et al. (2025) describe mitochondrial optic neuropathies, including m.3243A>G-related disease, as causing progressive RNFL thinning with preserved outer retinal layers, whereas our patient exhibited prominent outer retinal and photoreceptor involvement with intact scotopic function, effectively excluding mitochondrial and primary optic nerve disorders [16].

The lack of family history in this case suggests a sporadic form of cone dystrophy, a phenomenon documented in prior genetic studies, including Georgiou et al. [13]. Genetic confirmation may enhance diagnostic precision and prognostic evaluation; however, the clinical and electrophysiological profile was sufficient to establish the diagnosis.

This case illustrates how isolated cone dystrophy can masquerade as retinal vascular insufficiency and optic atrophy, particularly when asymmetrical and lacking classic symptoms. Comparison with recent genetic, imaging, and electrophysiological studies highlights the essential role of ERG and multimodal imaging in avoiding misdiagnosis. Recognizing such masquerades is crucial to prevent unnecessary vascular or neurological interventions, provide accurate counseling, and plan appropriate long-term visual rehabilitation and follow-up.

## Conclusions

This case study underscores the critical importance of considering isolated cone dystrophy in patients who present with painless, progressive visual impairment, particularly in situations where fundoscopic findings closely resemble those observed in vascular or optic neuropathic disorders. Accurate and timely diagnosis requires a thorough, comprehensive evaluation that incorporates multimodal imaging and detailed ERG to reliably distinguish cone dysfunction from other retinal or optic nerve pathologies. Early recognition of this condition is essential, as it not only prevents potential misdiagnosis and unnecessary medical or surgical interventions but also enables clinicians to provide patients with appropriate and timely counseling regarding prognosis, visual function, and management strategies. Moreover, heightened awareness of this rare and diagnostically challenging presentation empowers clinicians to make informed and precise clinical decisions, thereby contributing significantly to improved long-term visual outcomes and overall care for affected individuals.
